# Increased LC PUFA Levels in the Serum of Pregnant Women and Their Children as a Result of Dietary Supplementation with ‘Omega’ Fatty Acids

**DOI:** 10.3390/nu15010231

**Published:** 2023-01-02

**Authors:** Magdalena Broś-Konopielko, Agnieszka Białek, Monika Johne, Krzysztof Czajkowski

**Affiliations:** 1 II Faculty and Clinic of Obstetrics and Gynaecology, Medical University of Warsaw, Karowa 2, 00-315 Warsaw, Poland; 2 Faculty of Medical and Health Sciences, University of Economics and Human Sciences in Warsaw, Okopowa 59, 01-043 Warsaw, Poland; 3 The Kielanowski Institute of Animal Physiology and Nutrition, Polish Academy of Sciences, Instytucka 3, 05-110 Jabłonna, Poland; 4 Faculty of Physical Education, Józef Piłsudski University of Physical Education in Warsaw, Marymoncka 34, 00-968 Warsaw, Poland

**Keywords:** newborn, nutrition, diet, fatty acids, pregnancy, omega supplements, dietary supplements

## Abstract

Essential fatty acids (EFA) and long-chain polyunsaturated fatty acids (LC PUFA) are considered the most valuable bioactive fatty acids (FA) of the greatest importance for the mother’s and child’s health (e.g., placentation process, labor course, development of the central nervous system, visual acuity, cognitive functions), which results in dietary recommendations concerning EFA and LC PUFA intake in the diet of pregnant women. In this study, we aimed to evaluate the frequency of different food products consumption and ‘omega’ dietary supplements usage in groups of pregnant women. We also measured *n*-3 and *n*-6 FA content in serum samples of pregnant women and their children with the GC-FID technique, estimated the efficacy of applied supplementation, and compared the usefulness of different dietary supplements dedicated for pregnant women. ‘Omega’ dietary supplements effectively increased LC PUFA in the maternal blood (EPA, *p* = 0.0379; DHA *p* < 0.0001; *n*-3 PUFA, *p* < 0.0001), which penetrated the umbilical cord (EPA, *p* = 0.0131; DHA, *p* = 0.0288). If fish and seafood consumption is not enough, dietary supplements of the highest quality may provide sufficient LC PUFA without apprehension of MetHg contamination. ‘Omega’ dietary supplementation seems the most efficient way of providing an optimal supply of LC PUFA for the developing child from the earliest stages of development, which will bring advantages in the child’s future life and its health.

## 1. Introduction

Nutrition is one of most important environmental factors having strict life-long effects on health throughout life. The Developmental Programming hypothesis (also known as Developmental Origins of Health and Disease (DOHaD) hypothesis) emphasizes the crucial importance of maternal nutrition and maternal nutritional status as factors shaping the intrauterine and early postnatal environment of offspring. Developmental adaptations of progeny to nutritional signals from the mother are a normal part of development and adaptation to the future environment. Especially, the first 1000 days of life are claimed to be crucial for linking maternal nutrition to metabolic traits in offspring and in creating long-term health implications in children [1,2,3].

Following dietary recommendations by pregnant women is expected to fulfil all the nutritional requirements of a mother and a child. It is especially important during the period of the first 1000 days of life, which is a critical window in shaping the metabolism of a developing organism as well as the risk of some chronic diseases. Special attention is given to the adequate supply of essential fatty acids (EFA), which are precursors for long chain polyunsaturated fatty acids (LC PUFA). EFA include c9,c12 C18:2 (linoleic acid, LA), which is a precursor of the *n*-6 fatty acids (FA) family (including c6,c9,c12 C18:3 (γ-linolenic acid, GLA), and c5,c8,c11,c14 C20:4 (arachidonic acid, AA)), and c9,c12,c15 C18:3 (α-linolenic acid, ALA), which is a precursor of the *n*-3 FA family consisting of c5,c8,c11,c14,c17 C20:5 (eicosapentaenoic acid, EPA) and c4,c7,c10,c13,c16,c19 C22:6 (docosahexaenoic acid, DHA). LA and ALA compete for the same enzymes necessary for LC PUFA metabolism, although their metabolites have different functions and are not interchangeable. AA and EPA released from cellular membranes through the action of cyclooxygenase or lipoxygenase are transformed into series 2 and 3 prostanoids, respectively. Prostaglandins, prostacyclins, thromboxanes, and leukotrienes derived from LC PUFA play a key role in modulating inflammation, cytokine release, immune response, platelet aggregation, vascular reactivity, thrombosis, and allergic phenomenon. Series 3 prostanoids are weak agonists, or in some cases antagonize the activity of series 2 prostanoids. Eicosanoids of the 2 series promote inflammation, platelet aggregation and activate the immune response. In contrast, series 3 prostanoids tend to ameliorate these effect [4]. 

However, the endogenous synthesis of LC PUFA is insufficient regarding the needs of developing organism, and proper dietary supply of LC PUFA for pregnant women is of utmost importance. A meta-analysis by Abdelrahman et al. (2022) revealed that diet supplementation with *n*-3 LC PUFA in pregnancy can prevent preeclampsia, increase gestational duration, increase birth weight, and decrease the risk of low birth weight and preterm birth [5]. 

EFA and LC PUFA are considered the most valuable bioactive FA of the greatest importance for the health of both mother and child, which results in dietary recommendations concerning EFA and LC PUFA intake for pregnant women. Regarding EFA, there are only Australian recommendations. The daily ALA requirement for pregnant women is 1 g, and for lactating women 1.2 g, whereas the daily LA requirement for pregnant women is 10 g, and for lactating women is 12 g. Regarding LC PUFA, according to Australian recommendations, the total daily requirement for *n*-3 LC PUFA (DHA/EPA/DPA) is 0.110 g for pregnant women aged 14–18 years, 0.115 g for pregnant women over 19 years, and for lactating women it is 0.140–0.145 g [6]. The Polish Society of Gynaecologists and Obstetricians recommends dietary supplementation with 0.2 g DHA for pregnant woman. Moreover, in women with a low fish intake, the daily dose of supplemented DHA should increase to 0.6 g whereas in women at risk of preterm labor, the recommended dose of DHA should be even higher at 1.0 g per day [7]. Meanwhile, the Polish Paediatric Society recommends a combined dose of EPA+DHA at 1–1.5 g/day [8]. The upper limit of the maximum daily dose has not been determined, but research indicates that the supply of DHA up to 1.2 g and a total of 2.7 g of *n*-3 PUFA are safe [9]. According to other sources, the combined daily requirement for EPA+DHA for non-pregnant adults is 0.65 g, of which EPA is at least 0.22 g a day [10]. The maximum daily dose defined by the Australian recommendation has been determined as 3 g a day [6]. Fish and seafood are considered as the main dietary sources of *n*-3 LC PUFA and most dietary guidelines recommend 2–3 seafood meals per week. However, in many countries, the diets of pregnant women do not fulfil these recommendations. Many women are avoiding these food products as they may be sources of methylmercury (MeHg), which is neurotoxic, and even mildly elevated exposure on this compound during gestation can be harmful for the developing brain of the offspring [11]. Because of the small dietary intake of fish and seafood in the general population of Poland, which results in small supply of LC PUFA, it is considered that pregnant women, who consume small amounts of fish, ought to obtain 0.5–0.6 g of DHA a day in their diet from the onset of pregnancy. In complicated pregnancies, threatened with premature delivery, DHA supplementation ought to be even greater, amounting to 1 g DHA a day [12]. 

There are many dietary supplements dedicated for pregnant women and some of them are complex, multicomponent preparations containing vitamins, minerals, and other bioactive components, including LC PUFA. They are recommended to enrich the diet and provide an optimal intake of nutrients to the mother and her developing child, but also as a way of improving the health care regime for mothers and the progeny. Their composition, doses of active components, pharmaceutical forms, and price are diversified. However, solid data concerning the comparison of efficacy of different dietary supplements, especially in pregnant women, are limited. The first aim of this study was to evaluate the frequency of different food products consumption, mainly dietary fat sources, as well as ‘omega’ dietary supplements usage in groups of pregnant patients. The second objective was to measure, at the time of labor, the *n*-3 and *n*-6 FA content in serum samples of pregnant women and their children to understand of perinatal and infant nutritional status, also regarding the usage of ‘omega’ dietary supplements. We also try to estimate (as third objective of this study) the efficacy of applied supplementation and compare the usefulness of different dietary supplements dedicated for pregnant women.

## 2. Materials and Methods

### 2.1. Characteristics of Patients

Pregnant women that were patients of Anna Mazowiecka Clinical Hospital, who gave conscious written consent, received questionnaires concerning their diet, dietary habits, and nutritional status prior to labor. The questionnaire was drawn up together with the Department of Bromatology of the Faculty of Pharmacy with the Laboratory Medicine Division of the Warsaw Medical University. It enabled us to assess the diet of a pregnant women (especially the consumption of various food products, mostly being dietary sources of fat). The questions referred to the consumption statement (defined as “yes” or “no”) and frequency (defined as “never“, “once every 2 weeks“, “once a week“, “2–3 times a week“, “4–6 times a week“, and “everyday“) of consuming the food products and their estimated quantity/mass per helping. It also included questions concerning intake of omega 3 dietary supplements during pregnancy (“yes” or “no”) and the brand name of applied dietary supplement. The validity of the questionnaire was evaluated previously in pilot studies on group of 30 pregnant women (data not published) and was confirmed in previous studies. The study was conducted in the II Faculty and Clinic of Obstetrics and Gynecology of Medical University of Warsaw. It was approved by the Bioethics Commission of Medical University of Warsaw (KB 158/2010) and has been performed in accordance with the ethical standards laid down in the 1964 Declaration of Helsinki and its later amendments. 

At the beginning of pregnancy and before labor, the height and weight of the mothers were measured with the digital column scale, equipped with a stadiometer. It served for body mass index (BMI) assessment according to the standard formula: BMI = body mass (kg)/height (cm)^2^. At labor, the neonate’s height, weight, head circumference, and chest circumference were measured. Anthropometric, clinical, and pathological data for the studied patients are presented in Table 1. From a total of 161 women recruited for the study, six did not provide information concerning ‘omega’ 3 supplementation and they were excluded from the study. Serum samples were obtained from all 155 pregnant women and the fatty acids profile was investigated for all. Completed dietary questionnaires were obtained from 56 patients of the supplemented subgroup and 78 patients of the non-supplemented subgroup.

### 2.2. Fatty Acids Analysis

For the assessment of serum *n*-3 and *n*-6 FA content, maternal venous blood samples and umbilical cord blood samples were collected at delivery. Whole blood was collected into clot tubes and centrifuged. Separated serum samples were stored at −80 °C until FAs were analyzed. FA analysis was performed with gas chromatography (GC) using gas chromatograph (GC-17A gas chromatograph, Shimadzu, Kyoto, Japan) equipped with a capillary column (BPX 70; 30 m × 0.25 mm i.d., film thickness 0.20 μm, SGE, Ringwood, Australia) and a flame-ionization detector. Helium was the carrier gas. The initial oven temperature was 140 °C for 5 min, thereafter, it increased by 4 °C/min to 240 °C. The injector was heated to 250 °C and the detector to 270 °C. Fatty acids methyl esters (FAME) standards: methyl linoleate, methyl linolenate, methyl γ-linolenate, methyl arachidonate, methyl 5,8,11,14,17-eicosapentaenoate, 4,7,10,13,16,19-docosahexaenoic acid methyl ester, and Supelco 37 Component FAME Mix (Sigma-Aldrich, St. Louis, MO, USA) were used to identify the FA and/or prepare the standard curves. Standard curves were prepared with the use of hexane solutions of FAME of various concentrations, inserted to the column under the conditions described above. 

Standard curves (y = ax + b):

*n*-6 PUFA:

LA: y = 255,570x + 11860; R^2^ = 0.9933

GLA: y = 222,069x + 896.29; R^2^ = 0.9957

AA: y = 248,265x + 7052; R^2^ = 0.9944

*n*-3 PUFA:

ALA: y = 255,953x + 1486.8; R^2^ = 0.9956

EPA: y = 345,394x + 640.75; R^2^ = 0.9974

DHA: y = 219,176x − 3404; R^2^ = 0.9992

The serum samples were thawed only once and three parallel samples of 100 μL were trans-esterificated according to the procedure of Bondia-Pons et al. [13] with minor modifications. The serum samples were hydrolyzed without prior lipid extraction and fatty acids were converted to methyl esters by heating for 10 min with 2.5 mL sodium methoxide in methanol (0.5 mol/L) at 80 °C and afterwards by heating with 2.5 mL of 14% boron trifluoride-methanol reagent at 80 °C for 3 min. FAME were isolated with hexane (2 × 0.5 mL) after adding 1.0 mL of saturated sodium chloride solution. Organic extracts were dried with anhydrous sodium sulfate and evaporated to dryness under a stream of nitrogen. FAME were diluted in 50 μL (serum sample of mother) or in 20 μL (serum sample of child) and stored at −20 °C until being analyzed. One-microliter of analytical sample was subjected into the column. Three parallel samples were prepared from each serum sample and concentration of each individual FA in serum sample was quantified based on standard curves [µg/mL]. The percentage share of selected individual FAs in the total FA pool in the maternal serum sample was quantified by comparing the peak area of a given FA to the sum of the peak areas of all FAs present in the serum sample [%].

### 2.3. Statistical Analysis

For continuous variables, means and standard deviations were calculated. Categorical variables were described using frequencies and percentages. For variables with normal distribution (checked with Sahpiro-Wilk test), significant differences between supplemented and non-supplemented patients were established with the Student’s t test (marked * in Table 2), and among patients using different ‘omega’ dietary supplements, with a one-way ANOVA. For variables that did not meet the criteria of the normal distribution, a non-parametric test (Mann-Whitney U test) was applied for comparison of groups of supplemented and non-supplemented patients, whereas for comparison of patients using different ‘omega’ dietary supplement, the Kruskal-Wallis test was applied. All statistical analyses were performed using Statistica 13.0 (StatSoft Polska) statistical software. *p* < 0.05 was considered significant.

## 3. Results

### 3.1. Characteristics of Patients

Descriptive analysis was performed for the whole study population (cases) as well as for two revealed subgroups of supplemented and non-supplemented patients (Table 1). The mean age of women participating in the study was 31.2 ± 4.3 years and the mean height was 166.7 ± 6.1 cm. The mean body weight of investigated patients at the beginning of the pregnancy was 63.0 ± 11.8 kg, which resulted in a mean BMI of 22.6 ± 3.8 kg/m^2^. At the time of delivery, the mean body weight was 77.0 ± 13.1 kg, which resulted in a mean BMI of 27.6 ± 4.2 kg/m^2^. At the beginning of pregnancy, the BMI of most of women was correct (66.5%). Most women, 78.1%, had a higher education, 17.4% had a secondary education, and only 3.2% and 1.3% had a vocational and elementary education, respectively. None of investigated patients declared tobacco smoking and none of them claimed alcohol usage during pregnancy. A correct course of pregnancy was recorded in 63.2% of patients and 96.8% of them delivered on or after the 37th week of pregnancy. For most of the patients, it was first or second pregnancy, and only two patients delivered their fifth child; 31 patients experienced miscarriages previously. 

Among the investigated patients, 40% used ‘omega’ dietary supplements (*n* = 62). No differences in age, height, or week of delivery were observed between supplemented and non-supplemented patients. The mean weight of women applying ‘omega’ supplementation was significantly lower than that of women not using ‘omega’ supplementation. It resulted also in significantly lower (*p* = 0.0255) BMI in this group of patients. At labor, the final body mass of supplemented women was significantly lower than in non-supplemented women (*p* = 0.0227). Although no significant differences in final BMI values were revealed, the tendency for BMI values being lower in the subgroup of supplemented women was observed. Great similarity for most of the investigated variables were observed for subgroups of supplemented and non-supplemented women; however, some dependencies were also revealed. Among non-supplemented patients, 24.7% were overweight and 9.7% suffered from first stage obesity before pregnancy, respectively. 

Among the neonates, there were slightly more girls, 83 (53.5%), than boys, 72 (46.5%). The condition of the newborns after the 10th minute of life according to the Apgar scale was determined as good for 100% [14]. The majority of the newborns (81.3%) were healthy. The mean infant’s weight in the group of children whose mothers were subjected for ‘omega’ supplementation during pregnancy was not significantly different than in the case of children of non-supplemented mothers (3392 ± 409 g versus 3509 ± 448 g, *p* = 0.4583). Other parameters also did not differ between those two groups of infants. 

### 3.2. Diet Analysis

Completed questionnaires obtained from the patients (*n* = 133) were used for the analysis of diet during pregnancy, mainly regarding the dietary fat consumption. Dietary habits of investigated patients were compared due to declared omega supplementation. Detailed data are presented in Appendix A (Table A1, Table A2, Table A3, Table A4, Table A5, Table A6 and Table A7).

Olive and rapeseed oil were the most popular in the diet of investigated patients (Table A1). Only 22.6% of the supplemented subgroup and 30.1% of the non-supplemented subgroup totally excluded olive oil from their diet. The frequency of olive oil consumption in both subgroups was similar and each day olive oil was consumed by 16.1% of the supplemented and 12.9% of the non-supplemented patients. Patients using omega supplementation more willingly reached for rapeseed oil; 29% used it 1–3 times a week and 9.7% used it 4–6 times a week. Sunflower oil was much less popular and 54.8% of supplemented and 46.2% of non-supplemented women totally excluded it from their diets during pregnancy. Most of the pregnant women never used linseed oil in their diet. Similarly, soy, sesame, and grapeseed oils were only occasionally incorporated into the diet of both investigated subgroups whereas corn, coconut, peanut, and hemp oil were not used at all.

Butter was the most popular spreadable fat among pregnant women and each day it was consumed by 45.2% of supplemented and 33.3% of non-supplemented patients (Table A2). Margarine consumption was similar in the supplemented and non-supplemented groups of patients. However, it is alarming that 24.2% of the supplemented and 19.4% of the non-supplemented pregnant women ate this fat every day. Soft margarine or butter-margarine mixes were excluded from the diets of most of investigated pregnant patients. Also, similarities in lard or mayonnaise consumption patterns were observed between supplemented and non-supplemented groups of patients.

Generally, fish consumption was low in investigated patients. None of them incorporated fish in their diet daily (Table A3). Only 9.7% of supplemented and 3.2% of non-supplemented patients reached for salmon 2–3 times a week, and as many as 38.7% of supplemented and 50.5% of non-supplemented patients totally excluded it from their diet. Similar tendencies were observed for tuna intake wherein it was totally excluded from diet in 71.0% and 68.8% of supplemented and non-supplemented patients, respectively. Other types of fish were only occasionally consumed by our investigated patients, irrespectively from ‘omega’ supplementation.

The consumption patterns of nuts were similar in both investigated groups of patients (Table A4). Hazelnuts were the most popular in the diet of our patients; 9.8% of supplemented and 2.2% of non-supplemented patients reached for them each day. Similar popularity was observed for almonds, which were consumed every day by 4.8% of supplemented and 3.2% of non-supplemented women while they were excluded from the diet by 46.8% of supplemented and 58.1% of non-supplemented patients.

Eggs were excluded from diet by a very small percentage of pregnant patients; 4.8% of supplemented and 3.2% of non-supplemented patients (Table A5). The highest frequency of eggs consumption, which is 2–3 times a week, was reported for 40.3% of supplemented and 23.7% of non-supplemented patients, respectively.

Oatmeal was more popular in supplemented patients, as only 29.0% of them totally excluded it from diet, whereas in case of non-supplemented patients, this percentage was 50.5% (Table A6). Regarding cornflakes and rice places, no differences in consumption frequency patterns were observed. Sunflower seeds were an occasional snack for 29.0% of supplemented and 17.2% of non-supplemented patients, whereas pumpkin seeds were occasionally consumed by 16.1% of supplemented and 10.8% of non-supplemented pregnant women. About 75% of all investigated patients totally excluded wheat bread. Rye bread was more popular in the diet of pregnant women and 33.9% of supplemented women and 19.4% of non-supplemented women reached for it every day. Similarly, whole wheat bread was included daily in the diet of 27.1% of supplemented and 16.1% of non-supplemented pregnant women.

Frequency of salad and spinach consumption was higher in the subgroup of supplemented pregnant women whereas avocado consumption was similar (Table A7). Only two of the patients using ‘omega’ dietary supplements occasionally reached for tofu. Consumption of fries and chips were similar in the subgroups of supplemented and non-supplemented women. Most of the ‘omega’ supplemented women (83.9%) also used vitamin and mineral diet supplementation. One of the reasons is the fact that complex dietary supplements, containing both omega fatty acids and vitamins and minerals, were the most popular among pregnant women. Half of the ‘omega’ non-supplemented women resigned from vitamin-mineral diet supplementation during pregnancy.

### 3.3. FA Profile of Maternal Serum

The overall profile of FAs in maternal serum samples is given in Table 2. In the present experiment, 24 FAs were analyzed, including 10 saturated fatty acids (SFA), six monounsaturated fatty acids (MUFA), and eight polyunsaturated fatty acids (PUFA). Total shares of SFA, MUFA, and PUFA were comparable. Palmitic (C16:0), oleic (c9 C18:1, OL), LA, stearic (C18:0), and AA were the main FAs present in maternal serum samples. When comparing the FA profile of serum samples obtained from supplemented and non-supplemented patients, significant differences were revealed for three SFA (C15:0, C17:0, and C20:0), one MUFA (C20:1), and two PUFA (EPA and DHA). Applied ‘omega’ supplementation resulted in an increased concentration of those mentioned FAs. No significant differences in total SFA, MUFA, and PUFA share were observed between supplemented and non-supplemented patients. 

In relation to *n*-6 FA present in the serum, LA constituted about 18.9% of all FAs whereas AA, GLA, and C20:2 amounted to 4%, 0.18%, and 0.17%, respectively. Applied supplementation did not influence the percentage of particular *n*-6 FA share in the total FA pool, as well as the total *n*-6 PUFA share in the serum of supplemented and non-supplemented subgroups of patients (Table 2).

Regarding *n*-3 PUFA, the mean percentage share of ALA in the total FA pool in maternal serum was 0.6% and their metabolites constituted 0.31% (EPA) and 1.7% (DHA), respectively. Diet enrichment with ‘omega’ dietary supplements tended to increase ALA (*p* = 0.0894) and significantly increased the share of its metabolites EPA (*p* = 0.0379) and DHA (*p* < 0.0001) in the total FA pool in maternal serum. It resulted in a significantly higher total *n*-3 PUFA share in the total FA pool (*p* < 0.0001), as well as in a significantly lower (*p* = 0.0001) *n*-6/*n*-3 PUFA ratio in the serum of ‘omega’ supplemented patients (Table 2).

### 3.4. FA Content in the Serum of Mothers

In serum samples obtained by centrifugation of maternal venous blood samples collected at delivery into clot tubes, the concentration of *n*-6 and *n*-3 EFA (LA and ALA, respectively) as well as their main metabolites (*n*-6: GLA and AA and *n*-3: EPA and DHA) was quantified based on standard cures (Table 3). In the serum of mothers, LA predominated among *n*-6 PUFA with 10 times lower levels of AA and 100 times lower levels of GLA. The mean level of ALA, which is a precursor of *n*-3 PUFA family, was 26.0 ± 15.2 µg/mL, and no differences between subgroups of supplemented and non-supplemented patients were observed. Among its metabolites predominated DHA, whereas levels of EPA were 10 times lower. Applied supplementation significantly increased the DHA concentration in maternal blood (*p* = 0.0209) and tended to increase the EPA concentration as well (Table 3).

### 3.5. FA Content in Serum from Umbilical Cord Blood

Levels of *n*-6 and *n*-3 FAs in umbilical cord blood were from several to several dozen times lower than that in maternal venous blood (Table 3). Among *n*-6 PUFA, AA predominated with subsequent LA, with levels that were slightly lower than AA and GLA, which had concentrations 40 times lower than AA and LA. Applied supplementation did not influenced the amount of *n*-6 PUFA in umbilical cord blood.

DHA was the main *n*-3 PUFA detected in cord blood whereas EPA levels were 20 times lower than DHA. ALA concentrations were the lowest among all *n*-3 PUFA. Enrichment of maternal diet with ‘omega’ dietary supplements significantly increased EPA (*p* = 0.0131) and DHA (*p* = 0.0288) levels in umbilical cord blood, which increased the availability of EPA and DHA for infants.

### 3.6. ‘Omega’ Dietary Supplements

Among pregnant women taking part in this study, 62 (40%) of them used ‘omega’ dietary supplements. They reported usage of 15 different ‘omega’ dietary supplements of different brands. Their precise composition, declared by manufacturers, has been given in Table 4. Most of them contained solely *n*-3 FA (their amount in one capsule has been declared), for one supplement, containing fish oil, the exact amounts of EPA or DHA have not been declared (K), one supplement contained both declared amounts of EPA and DHA and *n*-6 PUFA (J) as well and one applied dietary supplement has only the *n*-6 PUFA amount declared (H). Among applied dietary supplements, four contained only *n*-3 PUFA (G, H, K, N), in three other *n*-3 PUFA were accompanied by vitamin E (F, J, O), mainly as antioxidants. The other seven supplements (A, B, C, D, E, I, L, M) were complex preparations, containing also different vitamins and minerals, in different amounts. The content of DHA in one capsule differed significantly among preparations, ranging from 20 mg DHA (in K) to 300 mg DHA (in F and J). Similarly, EPA content ranged from 30 mg (in B and C) to 330 mg (in O).

Investigated pregnant women most willingly used preparation A (14 persons—22.5%) and preparation B (12 persons—19.4%), whereas other dietary supplements were less popular (Table 4). Moreover, six of 62 patients (9.7%) decided to use two different ‘omega’ dietary supplements, mainly combining single ‘omega’ preparation with one of a complex preparation. No differences in *n*-6 and *n*-3 PUFA concentrations in maternal and in infantile serum were observed, irrespective of the applied supplement.

## 4. Discussion

Pregnancy is a period of intensive changes in a woman’s body, which relates to special dietary requirements. It is recommended that during pregnancy, some nutrients should be supplied in increased quantities while others should be entirely eliminated [15]. Dietary fats provide energy for growth, but also supply EFA, precursors of LC PUFA, which are considered the most valuable bioactive FA of the greatest importance for mother and child’s health. As the endogenous synthesis of LC PUFA is insufficient regarding the needs of a developing organism, proper supply of LC PUFA, especially EPA and DHA, during pregnancy is of utmost importance for labor course and the health of the child and mother. During the early period of pregnancy, LC PUFA derived from maternal diet and endogenous synthesis are stored in maternal adipose tissue. Subsequently, during the late period of pregnancy, lipid catabolism is enhanced due to the insulin-resistant condition, which causes the development of maternal hyperlipidemia. This phenomenon plays a key role in the availability of LC PUFA to the fetus [16]. In the first trimester, DHA is involved in placentation process by stimulating tube formation [17]. Placental vascular network is essential for the growth and maintenance of the developing embryo and LC PUFA of *n*-3 and *n*-6 families are directly or indirectly involved in angiogenesis. Metabolites of *n*-3 LC PUFA attenuate excess vascularization whereas the *n*-6 LC PUFA have a stimulatory or neutral effect on angiogenic processes [16]. Alterations in LC PUFA metabolites result in inadequate spiral artery remodeling or placental angiogenesis. Those structural and functional deficiencies of the placenta increase the risk of pregnancy complications, such as preeclampsia, gestational diabetes mellitus, intrauterine growth restriction, and results in adverse birth outcomes [17]. LC PUFA, especially DHA, plays a pivotal role in the development of the central nervous system, visual acuity, and cognitive functions. It depends on the involvement in maintaining membrane fluidity, impulse propagation, synaptic transmission, and functioning as a cytosolic signal-transducing factors for various gene expression during the critical period of brain development, which seems to be last trimester and first few months after birth. The highest accumulation of DHA by the fetus takes place during the third trimester of pregnancy and during the first years of life. DHA is accumulated mainly in the brain and retina [18]. Fetal brain growth is at its peak velocity during last trimester and first few months after birth, which makes the third-trimester fetus and newborn baby particularly vulnerable to LC PUFA deficits. As confirmed by a meta-analysis of numerous studies, preeclampsia, increased gestational duration, increased birth weight, and risk of low birth weight and preterm birth may be prevented by diet supplementation with *n*-3 LC PUFA during pregnancy [5]. Other authors also showed that an increased supply of *n*-3 FA results in slightly longer pregnancy duration, higher birth weight of the infant, reduction of the risk of premature birth, and better mental development during the first years of life [19,20,21,22].

Application of ‘omega’ dietary supplements, which was used by 40.0% of investigated women during pregnancy, has been proven as an efficient way to increase *n*-3 LC PUFA levels, both for mothers and for their children. Enrichment of the maternal diet with ‘omega’ dietary supplements significantly increased DHA content in their serum, comparing to those women who did not use ‘omega’ supplements, as well as EPA and DHA levels in umbilical cord blood. Our results confirmed usage of ‘omega’ dietary supplements as an efficient way of increasing the availability of EPA and DHA for infants. Other authors also indicated that supplementation with DHA preparations increased the concentration of maternal DHA in erythrocyte phospholipids, serum, and breast milk, as well as in umbilical blood [23,24]. Studies of Jackson et al. [25] also clearly showed that taking an EPA+DHA supplement increased the omega-3 index by 2.2% (*p* < 0.0001), which confirms that diet supplementation with omega-3 dietary supplements is an effective attitude. Kouba et al. observed that dietary supplementation of mares with marine-derived DHA or EPA/DHA during late gestation not only altered the fatty acids profile in the plasma of mothers, resulting in a greater concentration of EPA and DHA, but also resulted in *n*-3 LC PUFA transfer to the blood of dams [26]. According to review prepared by Mahaffey et al., it is possible to choose fish species that are both high in *n*-3 LC PUFAs and low in MetHg [27]. Moreover, it should be emphasized that as women are recommended to eat fish but to avoid mercury-containing fish, usage of ‘omega’ dietary supplements made from algae can provide a good solution due to the absence of MetHg contamination [23]. 

A review of randomized control trials made by Chmielewska et al. did not confirm a clear and consistent benefit of *n*-3 LC PUFA supplementation during pregnancy and/or lactation on childhood cognitive and visual development [28]. On the other hand, a review of human studies made by Ryan et al. clearly confirmed the efficiency of maternal LC PUFA supplementation during pregnancy and/or lactation and its relationship to childhood neurodevelopment, which appeared to be more effective than LC PUFA supplementation during childhood [29]. It is in accordance with meta-analysis of Qawasmi et al., who confirmed that LC PUFA supplementation of infant formulas did not exert any significant effect on improving early infant cognition [30]. Moreover, a randomized and double-blinded study by Helland et al. showed that children’s mental processing scores at 4 years of age correlated significantly with maternal intake of DHA and EPA during pregnancy. They also revealed, in a multiple regression model, that maternal intake of DHA during pregnancy was the only variable of statistical significance for the children’s mental processing scores at 4 years of age [20]. 

LC PUFA are considered as pivotal for mental status. A meta-analysis of trials conducted on patients with a defined diagnosis of major depressive disorder and patients with depressive symptomatology but no diagnosis of major depressive disorder, performed by Grosso et al., confirmed the effectiveness of *n*-3 LC PUFA. Use of mainly EPA within the preparation, rather than DHA, as well as use of *n*-3 LC PUFA as adjuvant rather than monotherapy, influenced the final clinical efficacy [31]. Also, the results of a pairwise meta-analysis by Luo et al. showed that high-dose *n*-3 LC PUFA supplementation might be more superior than low-dose in the early therapy period for major depressive disorder, although both the high and the low-dose *n*-3 LC PUFA were superior to placebo [32]. A meta-analysis performed by Wolters et al. indicated that *n*-3 LC PUFA supplementation lowered depressive symptoms as compared with placebo. Beneficial effects were seen in the subgroups of studies with longer treatment duration and with no depression and mild to moderate depression [33]. 

The potential mechanism by which LC PUFA (especially *n*-3 LC PUFA) exerts an antidepressant effect is their anti-inflammatory properties. Some of the subtypes of depression seem to be associated with inflammation and this association seems bidirectional. Physically ill patients with chronic inflammation often reveal the symptoms of depression. Many authors showed an association between concentrations of pro-inflammatory cytokines, specifically interleukins 1β (IL-1β) and 6 (IL-6), and tumor necrosis factor α (TNFα), and depressive symptoms. There is also evidence that this pro-inflammatory state is accompanied by aberrant inflammation-related processes, including platelet activation factor hyperactivity, oxidative and nitrosative stress, and damage to mitochondria. These complex and interrelated mechanisms can collectively contribute to negative neurobiological outcomes that may underlie the etiopathology of depression. Depression emerges on the background of sickness when the inflammatory response is too intense and long lasting, or the resolution process is deficient. A concomitant reduction in both depressive symptoms and pro-inflammatory cytokine concentrations resulting from treatment with pharmacological anti-inflammatory interventions was observed [34]. One of the main benefits of LC PUFA for depression treatment is their favorable safety profile and lack of adverse reactions, including reactions with concomitant medications, which is highly appropriate as a treatment of depression related to pregnancy but also for pediatric populations. One of the new approaches in treating depressive symptoms (especially if inflammation is involved, standard treatment is ineffective or disease affects specific vulnerable populations, such as pregnant women or children) is to use dietary supplements that demonstrate the ability to target inflammation and other underlying systems in depression, in particular, LC PUFA, probiotics, and folic acid. As shown by Krauss-Etschmann et al. [24], folate supplementation from gestation week 22 to birth significantly (*p* = 0.047) influenced the maternal DHA level. However, there is no consensus in research data concerning depression related to pregnancy and LC PUFA supply. Studies of Pinto et al. in low-income pregnant Brazilian women showed that lower serum concentrations of DHA, EPA, and DPA, and a higher *n*-6/*n*-3 ratio at each pregnancy trimester, were associated with higher odds of depressive symptoms throughout pregnancy [35]. A meta-analysis prepared by Suradom et al. with the pooled standardized mean of decreased depression scores revealed no statistically significant difference between the *n*-3 LC PUFA and the placebo groups and no statistically significant efficacy of *n*-3 LC PUFA supplementation for the prevention and treatment of perinatal depression [36]. The efficacy of *n*-3 LC PUFA supplementation was not associated with the daily doses of DHA, EPA, or DHA+EPA. A review of randomized controlled trials, performed by Miller et al., showed no sufficient evidence to conclude that selenium, DHA, or EPA prevented postnatal depression and no evidence to recommend any dietary supplement for the prevention of postnatal depression [37]. 

## 5. Conclusions

Omega’ dietary supplements dedicated for pregnant women effectively increase LC PUFA in the maternal blood, which can penetrate the umbilical cord. It seems the most efficient way of providing their optimal supply for the developing child from the earliest stages of its development, which will bring advantages in the childs future life and health. Although strong clinical evidence is still eligible, the available data confirms most of the benefits of adequate LC PUFA supply during pregnancy. If fish and seafood consumption is not enough, dietary supplements of the highest quality may provide sufficient amounts of LC PUFA without apprehension of MetHg contamination.

## Figures and Tables

**Table 1:** Characteristics of the participants.

Mother		All *n* = 155		Supplemented *n* = 62		Non-Supplemented *n* = 93		*p*-Value
Age [years]		31.2 ± 4.3		31.5 ± 4.4		31.0 ± 4.2		0.6859
Height [cm]		166.7 ± 6.1		167.0 ± 5.6		166.6 ± 6.5		0.2367
Body weight before pregnancy [kg]		63.0 ± 11.8		59.7 ± 9.1		65.2 ± 13.0		0.0034
Body weight on enrollment [kg]		77.0 ± 13.1		73.5 ± 10.7		79.3 ± 14.1		0.0227
BMI before pregnancy [kg/m^2^]		22.6 ± 3.8		21.4 ± 3.1		23.5 ± 4.0		0.0255
BMI on enrollment [kg/m^2^]		27.6 ± 4.2		26.3 ± 3.6		28.5 ± 4.3		0.1861
Week of delivery		38.9 ± 35.0		38.9 ± 1.3		38.9 ± 1.2		0.9064
		*n*	%	*n*	%	*n*	%	
Diseases before pregnancy	no	120	77.4	45	72.6	75	80.6	
	yes	35	22.6	17	27.4	18	19.4	
	no data	0	0.0	0	0.0	0	0.0	
Diseases during pregnancy								
	no	98	63.2	43	69.4	55	59.1	
	yes	57	36.8	19	30.6	38	40.9	
	no data	0	0.0	0	0.0	0	0.0	
Type of disease	GDMG1			10	16.1	17	18.3	
	GDMG1 + PIH without proteinuria			1	1.6	0	0.0	
	GDMG1 + cholestasis			1	1.6	1	1.1	
	PIH without proteinuria			0	0.0	3	3.2	
	PIH with proteinuria			1	1.6	0	0.0	
	proteinuria			2	3.2	0	0.0	
	PPH without proteinuria			0	0.0	3	3.2	
	cholestasis			1	1.6	5	5.4	
	PGDM			0	0.0	1	1.1	
	GDMG2			2	3.2	5	5.4	
	cholestasis + PPH without proteinuria			0	0.0	1	1.1	
	cholestasis + PGDM			0	0.0	1	1.1	
	toxo			0	0.0	1	1.1	
	intrauterine infection			1	1.6	0	0.0	
	no data			0	0.0	0	0.0	
Education								
	higher	121	78.1	54	87.1	67	72.0	
	secondary	27	17.4	7	11.3	20	21.5	
	elementary	2	1.3	0	0.0	2	2.2	
	vocational	5	3.2	1	1.6	4	4.3	
	no data	0	0.0	0	0.0	0	0.0	
BMI before pregnancy classification								
	starvation	0	0.0	0	0.0	0	0.0	
	emaciation	5	3.2	3	4.8	2	2.2	
	underweight	7	4.5	4	6.5	3	3.2	
	correct	103	66.5	47	75.8	56	60.2	
	overweight	30	19.4	7	11.3	23	24.7	
	1st degree obesity	10	6.5	1	1.6	9	9.7	
	2nd degree obesity	0	0.0	0	0.0	0	0.0	
	3rd degree obesity	0	0.0	0	0.0	0	0.0	
	no data	0	0.0	0	0.0	0	0.0	
Sequence number of pregnancy								
	1st	57	36.8	24	38.7	33	35.5	
	2nd	51	32.9	18	29.0	33	35.5	
	3rd	31	20.0	14	22.6	17	18.3	
	4th	14	9.0	5	8.1	9	9.7	
	5th	2	1.3	1	1.6	1	1.1	
	no data	0	0.0	0	0.0	0	0.0	
Number of previous deliveries								
	0	65	41.9	30	48.4	35	37.6	
	1	61	39.4	22	35.5	39	41.9	
	2	23	14.8	8	12.9	15	16.1	
	3	5	3.2	2	3.2	3	3.2	
	4	1	0.6	0	0.0	1	1.1	
	no data	0	0.0	0	0.0	0	0.0	
Number of miscarriages								
	0	124	80.0	46	74.2	78	83.9	
	1	25	16.1	12	19.4	13	14.0	
	2	4	2.6	2	3.2	2	2.2	
	3	2	1.3	2	3.2	0	0.0	
	no data	0	0.0	0	0.0	0	0.0	
Tobacco smoking during pregnancy								
	no	155	100.0	62	100.0	93	100.0	
	yes	0	0.0	0	0.0	0	0.0	
	no data	0	0.0	0	0.0	0	0.0	
Alcohol drinking during pregnancy								
	no	155	100.0	62	100.0	93	100.0	
	yes	0	0.0	0	0.0	0	0.0	
	no data	0	0.0	0	0.0	0	0.0	
Delivery								
	<37th week	5	3.2	3	4.8	2	2.2	
	≥37th week	150	96.8	59	95.2	91	97.8	
	no data	0	0.0	0	0.0	0	0.0	
Mode of delivery								
	CC	44	28.4	14	22.6	30	32.3	
	PNS	109	70.3	47	75.8	62	66.7	
	VE	2	1.3	1	1.6	1	1.1	
	no data	0	0.0	0	0.0	0	0.0	
Gender of child								
	female	83	53.5	32	51.6	51	54.8	
	male	72	46.5	30	48.4	42	45.2	
	no data	0	0.0	0	0.0	0	0.0	
**Child**								
Head circumference [cm]		34.6 ± 1.5		34.6 ± 1.3		34.6 ± 1.6		0.1763
Chest circumference [cm]		33.7 ± 1.8		33.5 ± 1.7		33.8 ± 1.8		0.3886
Newborn’s weight [g]		3462 ± 435		3392 ± 409		3509 ± 448		0.4583
Newborn’s body length [cm]		54.4 ± 2.9		53.8 ± 2.9		54.9 ± 2.9		0.9806
Newborn’s Ponderal Index [kg/m^3^]		21.6 ± 2.7		22.0 ± 2.7		21.4 ± 2.7		0.8772
Apgar 10′								
		*n*	%					
Apgar scoring								
	good (10–8)	155	100.0	62	100.0	93	100.0	
	average (7–4)	0	0.0	0	0.0	0	0.0	
	severe (3–0)	0	0.0	0	0.0	0	0.0	

**Table 2:** Fatty acids profile in maternal serum.

	All	Supplemented	Non-Supplemented	*p*-Value
Fatty Acid [%]	*n* = 155	*n* = 62	*n* = 93	
C12:0	0.09 ± 0.04	0.09 ± 0.03	0.09 ± 0.05	0.9577
C14:0	0.96 ± 0.29	0.96 ± 0.29	0.96 ± 0.30	0.8265
C15:0	0.22 ± 0.05	0.23 ± 0.06	0.21 ± 0.05	0.0036
C16:0	22.95 ± 1.99	22.60 ± 2.04	23.18 ± 1.94	0.0737
C17:0	0.20 ± 0.04	0.21 ± 0.05	0.20 ± 0.03	0.0120
C18:0	4.15 ± 0.54	4.16 ± 0.65	4.14 ± 0.46	0.7812 *
C20:0	0.04 ± 0.05	0.05 ± 0.06	0.03 ± 0.04	0.0262
C22:0	0.09 ± 0.04	0.09 ± 0.05	0.09 ± 0.04	0.0540
C23:0	0.06 ± 0.04	0.07 ± 0.05	0.06 ± 0.04	0.7395
C24:0	0.12 ± 0.05	0.12 ± 0.06	0.12 ± 0.03	0.0761
SFA	28.88 ± 2.25	28.58 ± 2.36	29.08 ± 2.16	0.1646
C14:1	0.04 ± 0.02	0.04 ± 0.02	0.04 ± 0.02	0.2491
C16:1	2.18 ± 0.73	2.23 ± 0.84	2.15 ± 0.66	0.9840
C17:1	0.16 ± 0.04	0.17 ± 0.05	0.16 ± 0.03	0.7576
c9 C18:1	20.42 ± 2.61	20.20 ± 2.40	20.56 ± 2.74	0.4049 *
C20:1	0.02 ± 0.01	0.03 ± 0.01	0.02 ± 0.01	0.0023
C22:1	0.02 ± 0.02	0.02 ± 0.02	0.02 ± 0.01	0.9200
MUFA	22.85 ± 2.70	22.69 ± 2.53	22.95 ± 2.82	0.2621 *
t9,t12 C18:2	0.07 ± 0.02	0.07 ± 0.03	0.07 ± 0.02	0.8882
LA	18.92 ± 2.69	18.99 ± 3.06	18.88 ± 2.44	0.3022
GLA	0.18 ± 0.10	0.19 ± 0.13	0.17 ± 0.07	0.5639
ALA	0.60 ± 0.19	0.64 ± 0.22	0.58 ± 0.17	0.0894
C20:2	0.17 ± 0.05	0.17 ± 0.05	0.16 ± 0.05	0.2987
AA	3.92 ± 0.79	3.88 ± 0.95	3.95 ± 0.67	0.3709
EPA	0.31 ± 0.27	0.37 ± 0.37	0.27 ± 0.17	0.0379
DHA	1.72 ± 0.41	1.89 ± 0.45	1.60 ± 0.33	<0.0001
PUFA	25.89 ± 2.89	26.21 ± 3.29	25.68 ± 2.58	0.1210
*n*-6 PUFA	23.26 ± 2.76	23.31 ± 3.13	23.23 ± 2.50	0.3937
*n*-3 PUFA	2.63 ± 0.67	2.90 ± 0.79	2.45 ± 0.50	<0.0001
*n*-6/*n*-3 PUFA	9.32 ± 2.30	8.50 ± 2.29	9.86 ± 2.16	0.0001

For variables with normal distribution (checked with Shapiro-Wilk test), significant differences between supplemented and non-supplemented patients were established with the Student’s t test (marked * in Table 2). For variables that did not meet the criteria of the normal distribution, a non-parametric test (Mann-Whitney U test) was applied for comparison of groups of supplemented and non-supplemented patients. All statistical analyses were performed using Statistica 13.0 (StatSoft Polska) statistical software. *p* < 0.05 was considered significant.

**Table 3:** Fatty acid content in serum samples of mothers and children.

Fatty Acids	All *n* = 155		Supplemented *n* = 62		Non-Supplemented *n* = 93		*p*-Value
	Mothers						
*n*-6 PUFA [µg/mL]							
mLA	957.5 ± 319.4		959.8 ± 294.5		956.0 ± 336.5		0.6865
mGLA	9.3 ± 9.8		10.7 ± 13.0		8.4 ± 6.9		0.7465
mAA	191.8 ± 6.4		185.5 ± 63.5		196.1 ± 65.1		0.2302
*n*-3 PUFA [µg/mL]							
mALA	26.0 ± 15.2		25.1 ± 14.7		26.6 ± 15.6		0.7996
mEPA	10.7 ± 11.4		13.0 ± 15.7		9.2 ± 7.1		0.0793
mDHA	107.4 ± 36.5		115.0 ± 38.2		102.3 ± 34.6		0.0209
	Children						
*n*-6 PUFA [µg/mL]							
cLA	83.9 ± 35.8		86.6 ± 40.4		82.1 ± 32.6		0.5359
cGLA	2.4 ± 1.3		2.5 ± 1.2		2.3 ± 1.4		0.1357
cAA	92.7 ± 31.2		89.7 ± 29.1		94.6 ± 32.6		0.5191
*n*-3 PUFA [µg/mL]							
cALA	1.4 ± 2.1		1.9 ± 2.9		1.1 ± 1.2		0.5452
cEPA	1.9 ± 1.3		2.2 ± 1.5		1.7 ± 1.2		0.0131
cDHA	38.6 ± 12.8		41.4 ± 14.0		36.7 ± 11.6		0.0288

**Table 4:** Detailed composition of applied omega dietary supplements.

Dietary Supplement	*n*-3 PUFA in 1 Capsule	*n*-6 PUFA in 1 Capsule	Recommended Intake	Other Components
A	200 mg DHA	not declared	1 caps/day	Vit. E 12 mg, Folates 0.6 mg (including: 0.2 mg folic acid, 0.416 mg calcium L-methylfolate), Vit. B1 1.5 mg, Vit. B2 1.6 mg, Vit. B6 2.2 mg, Vit. B12 2.7 µg, Biotin 0.1 mg, Niacin 20 mg, Pantothenic Acid 10 mg, β-carotene 3 mg, Vit. C 180 mg, Vit. D 10 µg, Magnesium 70 mg, Iron 28 mg, Zink 15 mg, Iodine 0.15 mg, Copper 1 mg, Manganese 1 mg
B	3300 mg: 150 mg DHA, 30 mg EPA	not declared	1 caps/day	Vit. C 85 mg, Vit. B3 18 mg, Vit. E 15 mg, Vit. B1 1.4 mg, Vit. B2 1.4 mg, Folic acid 600 µg, β-carotene 2 mg, Biotin 30 µg, Vit. D 5 µg, Vit. B12 2.6 µg, Iron 27 mg, Zink 11 mg, Magnesium 10 mg, Copper 1 mg
C	100 mg fish oil: 20 mg DHA, 30 mg EPA	not declared	1 caps/day	Iron 26 mg, Folic acid 600 µg (including 400 µg pteroylmonoglutamic acid), 208 µg calcium L-methylfolate, Vit. D 50 µg, Iodine 200 µg
D	160 mg DHA, 32 mg EPA	not declared	2 caps/day	Vit. C 80 mg, Niacin 18 mg, Vit. E 10 mg, Pantothenic acid 6 mg, Vit. B6 2 mg, Vit. B2 1.6 mg, Vit. B1 1.4 mg, Folic acid 400 µg, Vit. B12 2.6 µg, Vit. D3 10 µg, Biotin 50 µg, Magnesium 75 mg, Manganese 1 mg, Iron 27 mg, Zinc 10 mg, Iodine 200 mcg, Selenium 60 µg, Copper 1 mg
E	200 mg DHA, 43 mg EPA	not declared	1 caps/day	β-carotene 2 mg, Vit. E 12 mg, Vit. C 110 mg, Vit. B1 1.2 mg, Vit. B2 1.4 mg, Vit. B6 1.6 mg, Vit. B12 3 µg, Vit. D 5 µg, Biotin 100 µg, Folic acid 400 µg, Niacin 14 mg, Pantothenic acid 6 mg, Calcium 131 mg, Magnesium 100 mg, Iron 15 mg, Iodine 200 µg, Copper 1000 µg, Manganese 1 mg, Selenium 25 µg, Zinc 7 mg, Vit. E 12 mg
F	300 mg DHA, 35 mg EPA	not declared	1–2 caps/day	Vit. E 5.85 mg
G	200 mg DHA	not declared	1–2 caps/day	
H	not declared	Evening primrose oil including: 347.11 mg LA, 42.39 mg GLA	1–2 caps/2 times a day	
I	200 mg DHA	not declared	1 caps/day	Iron 27 mg, Iodine 150 µg, Vit. D3 20 µg
J	300 mg DHA, 42 mg EPA,	Evening primrose oil including: 15 mg GLA, 8.4 mg AA,	2 caps/day	Vit. E 2.8 mg
K	Fish oil 250 mg including 50 mg alkoxyglicerols	not declared	1–2 caps/day	
L	220 mg DHA, 44 mg EPA	not declared	1 caps/day	β-carotene 3 mg, Vit. D3 10 µg, Vit. E 10 mg, Vit. B1 1.66 mg, Vit B2 2.2 mg, Vit. B12 1.2 µg, Vit. C 100 mg, Niacin 17.8 mg, Biotin 150 µg, Folic acid 400 µg, Pantothenic acid 10.3 mg, Calcium 200 mg, Magnesium 50 mg, Zinc 15 mg, Iron 28 mg, Manganese 1 mg, Copper 1 mg, Iodine 150 µg, Selenium 20 µg
M	250 mg DHA	not declared	2 caps/day	Iron 30 mg, Vit. D 2000 i.u., Folate 0.8 mg, Iodine 0.2 mg
N	Fish oil: 27.5 mg DHA, EPA 42 mg	not declared	1 caps/day	
O	Fish oil: 220 mg DHA, 330 mg EPA, 100 mg other *n*-3 PUFA	not declared	1-2 caps/day	Vit. E 12 mg

## Data Availability

Raw data available on request.

## Appendix A

**Table A1:** Oils consumption among investigated patients.

		Supplemented *n* = 62		Non-Supplemented *n* = 93	
Olive		*n*	%	*n*	%
	everyday	10	16.1	12	12.9
	4–6 times a week	3	4.8	5	5.4
	2–3 times a week	13	21.0	11	11.8
	once a week	8	12.9	10	10.8
	once every 2 weeks	8	12.9	12	12.9
	never	14	22.6	28	30.1
	no data	6	9.7	15	16.1
Sunflower					
	everyday	3	4.8	3	3.2
	4–6 times a week	0	0.0	0	0.0
	2–3 times a week	3	4.8	16	17.2
	once a week	9	14.5	6	6.5
	once every 2 weeks	7	11.3	10	10.8
	never	34	54.8	43	46.2
	no data	6	9.7	15	16.1
Rapeseed					
	everyday	4	6.5	7	7.5
	4–6 times a week	6	9.7	3	3.2
	2–3 times a week	9	14.5	9	9.7
	once a week	9	14.5	4	4.3
	once every 2 weeks	4	6.5	4	4.3
	never	24	38.7	51	54.8
	no data	6	9.7	15	16.1
Linseed					
	everyday	1	1.6	0	0.0
	4–6 times a week	0	0.0	3	3.2
	2–3 times a week	1	1.6	0	0.0
	once a week	0	0.0	0	0.0
	once every 2 weeks	1	1.6	0	0.0
	never	53	85.5	75	80.6
	no data	6	9.7	15	16.1
Corn					
	everyday	0	0.0	0	0.0
	4–6 times a week	0	0.0	0	0.0
	2–3 times a week	0	0.0	0	0.0
	once a week	0	0.0	0	0.0
	once every 2 weeks	0	0.0	0	0.0
	never	56	90.3	78	83.9
	no data	6	9.7	15	16.1
Grapeseed					
	everyday	0	0.0	1	1.1
	4–6 times a week	1	1.6	0	0.0
	2–3 times a week	1	1.6	0	0.0
	once a week	0	0.0	1	1.1
	once every 2 weeks	0	0.0	2	2.2
	never	54	87.1	74	79.6
	no data	6	9.7	15	16.1
Coconut					
	everyday	0	0.0	0	0.0
	4–6 times a week	0	0.0	0	0.0
	2–3 times a week	0	0.0	0	0.0
	once a week	0	0.0	0	0.0
	once every 2 weeks	0	0.0	0	0.0
	never	56	90.3	78	83.9
	no data	6	9.7	15	16.1
Sesame					
	everyday	0	0.0	0	0.0
	4–6 times a week	0	0.0	0	0.0
	2–3 times a week	0	0.0	0	0.0
	once a week	1	1.6	0	0.0
	once every 2 weeks	0	0.0	0	0.0
	never	55	88.7	78	83.9
	no data	6	9.7	15	16.1
Soybean					
	everyday	0	0.0	0	0.0
	4–6 times a week	0	0.0	0	0.0
	2–3 times a week	0	0.0	0	0.0
	once a week	1	1.6	0	0.0
	once every 2 weeks	0	0.0	1	1.1
	never	54	87.1	77	82.8
	no data	7	11.3	15	16.1
Peanut					
	everyday	0	0.0	0	0.0
	4–6 times a week	0	0.0	0	0.0
	2–3 times a week	0	0.0	0	0.0
	once a week	0	0.0	0	0.0
	once every 2 weeks	0	0.0	0	0.0
	never	56	90.3	78	83.9
	no data	6	9.7	15	16.1
Hemp					
	everyday	0	0.0	0	0.0
	4–6 times a week	0	0.0	0	0.0
	2–3 times a week	0	0.0	0	0.0
	once a week	0	0.0	0	0.0
	once every 2 weeks	0	0.0	0	0.0
	never	56	90.3	78	83.9
	no data	6	9.7	15	16.1
Pumpkin					
	everyday	0	0.0	0	0.0
	4–6 times a week	0	0.0	0	0.0
	2–3 times a week	0	0.0	0	0.0
	once a week	0	0.0	0	0.0
	once every 2 weeks	0	0.0	0	0.0
	never	56	90.3	78	83.9
	no data	6	9.7	15	16.1

**Table A2:** Fats consumption among investigated patients.

		Supplemented *n* = 62		Non-Supplemented *n* = 93	
Margarine		*n*	%	*n*	%
	everyday	15	24.2	18	19.4
	4–6 times a week	1	1.6	4	4.3
	2–3 times a week	1	1.6	4	4.3
	once a week	1	1.6	0	0.0
	once every 2 weeks	0	0.0	1	1.1
	never	38	61.3	51	54.8
	no data	6	9.7	15	16.1
Soft margarine					
	everyday	1	1.6	6	6.5
	4–6 times a week	0	0.0	1	1.1
	2–3 times a week	0	0.0	1	1.1
	once a week	0	0.0	0	0.0
	once every 2 weeks	0	0.0	1	1.1
	never	55	88.7	69	74.2
	no data	6	9.7	15	16.1
Butter					
	everyday	28	45.2	31	33.3
	4–6 times a week	3	4.8	10	10.8
	2–3 times a week	7	11.3	5	5.4
	once a week	1	1.6	0	0.0
	once every 2 weeks	1	1.6	2	2.2
	never	17	27.4	30	32.3
	no data	5	8.1	15	16.1
Butter-margarine mix					
	everyday	2	3.2	1	1.1
	4–6 times a week	0	0.0	0	0.0
	2–3 times a week	0	0.0	2	2.2
	once a week	0	0.0	0	0.0
	once every 2 weeks	0	0.0	1	1.1
	never	54	87.1	74	79.6
	no data	6	9.7	15	16.1
Lard					
	everyday	0	0.0	0	0.0
	4–6 times a week	0	0.0	0	0.0
	2–3 times a week	1	1.6	2	2.2
	once a week	0	0.0	0	0.0
	once every 2 weeks	0	0.0	1	1.1
	never	55	88.7	75	80.6
	no data	6	9.7	15	16.1
Mayonnaise					
	everyday	1	1.6	1	1.1
	4–6 times a week	2	3.2	4	4.3
	2–3 times a week	3	4.8	7	7.5
	once a week	7	11.3	7	7.5
	once every 2 weeks	14	22.6	20	21.5
	never	29	46.8	39	41.9
	no data	6	9.7	15	16.1

**Table A3:** Fish consumption among investigated patients.

		Supplemented *n* = 62		Non-Supplemented *n* = 93	
Salmon		*n*	%	*n*	%
	everyday	0	0.0	0	0.0
	4–6 times a week	0	0.0	0	0.0
	2–3 times a week	6	9.7	3	3.2
	once a week	7	11.3	7	7.5
	once every 2 weeks	20	32.3	21	22.6
	never	24	38.7	47	50.5
	no data	5	8.1	15	16.1
Tuna					
	everyday	0	0.0	0	0.0
	4–6 times a week	0	0.0	0	0.0
	2–3 times a week	2	3.2	2	2.2
	once a week	1	1.6	5	5.4
	once every 2 weeks	10	16.1	7	7.5
	never	44	71.0	64	68.8
	no data	5	8.1	15	16.1
Mackerel					
	everyday	0	0.0	0	0.0
	4–6 times a week	0	0.0	0	0.0
	2–3 times a week	0	0.0	2	2.2
	once a week	5	8.1	2	2.2
	once every 2 weeks	14	22.6	23	24.7
	never	38	61.3	51	54.8
	no data	5	8.1	15	16.1
Trout					
	everyday	0	0.0	0	0.0
	4–6 times a week	0	0.0	0	0.0
	2–3 times a week	0	0.0	0	0.0
	once a week	2	3.2	1	1.1
	once every 2 weeks	6	9.7	7	7.5
	never	49	79.0	70	75.3
	no data	5	8.1	15	16.1
Cod					
	everyday	0	0.0	0	0.0
	4–6 times a week	0	0.0	0	0.0
	2–3 times a week	1	1.6	0	0.0
	once a week	3	4.8	5	5.4
	once every 2 weeks	15	24.2	18	19.4
	never	38	61.3	55	59.1
	no data	5	8.1	15	16.1
Herring					
	everyday	0	0.0	0	0.0
	4–6 times a week	2	3.2	0	0.0
	2–3 times a week	3	4.8	1	1.1
	once a week	2	3.2	5	5.4
	once every 2 weeks	13	21.0	12	12.9
	never	37	59.7	60	64.5
	no data	5	8.1	15	16.1
Sardine					
	everyday	0	0.0	0	0.0
	4–6 times a week	0	0.0	0	0.0
	2–3 times a week	0	0.0	0	0.0
	once a week	0	0.0	0	0.0
	once every 2 weeks	1	1.6	0	0.0
	never	56	90.3	78	83.9
	no data	5	8.1	15	16.1
Eel					
	everyday	0	0.0	0	0.0
	4–6 times a week	0	0.0	0	0.0
	2–3 times a week	0	0.0	0	0.0
	once a week	0	0.0	0	0.0
	once every 2 weeks	0	0.0	0	0.0
	never	57	91.9	78	83.9
	no data	5	8.1	15	16.1
Halibut					
	everyday	0	0.0	0	0.0
	4–6 times a week	0	0.0	0	0.0
	2–3 times a week	0	0.0	0	0.0
	once a week	0	0.0	0	0.0
	once every 2 weeks	3	4.8	0	0.0
	never	54	87.1	78	83.9
	no data	5	8.1	15	16.1
Sprat					
	everyday	0	0.0	0	0.0
	4–6 times a week	0	0.0	0	0.0
	2–3 times a week	0	0.0	0	0.0
	once a week	1	1.6	1	1.1
	once every 2 weeks	1	1.6	5	5.4
	never	55	88.7	72	77.4
	no data	5	8.1	15	16.1
Pollock					
	everyday	0	0.0	0	0.0
	4–6 times a week	0	0.0	0	0.0
	2–3 times a week	0	0.0	0	0.0
	once a week	1	1.6	0	0.0
	once every 2 weeks	1	1.6	1	1.1
	never	55	88.7	77	82.8
	no data	5	8.1	15	16.1

**Table A4:** Nuts consumption among investigated patients.

		Supplemented *n* = 62		Non-Supplemented *n* = 93	
Hazelnuts		*n*	%	*n*	%
	everyday	1	1.6	1	1.1
	4–6 times a week	1	1.6	2	2.2
	2–3 times a week	7	11.3	2	2.2
	once a week	1	1.6	7	7.5
	once every 2 weeks	15	24.2	14	15.1
	never	31	50.0	52	55.9
	no data	6	9.7	15	16.1
Walnuts					
	everyday	3	4.8	2	2.2
	4–6 times a week	0	0.0	0	0.0
	2–3 times a week	7	11.3	2	2.2
	once a week	2	3.2	8	8.6
	once every 2 weeks	17	27.4	16	17.2
	never	27	43.5	50	53.8
	no data	6	9.7	15	16.1
Cashews					
	everyday	0	0.0	0	0.0
	4–6 times a week	0	0.0	0	0.0
	2–3 times a week	4	6.5	3	3.2
	once a week	2	3.2	1	1.1
	once every 2 weeks	9	14.5	6	6.5
	never	41	66.1	68	73.1
	no data	6	9.7	15	16.1
pistachios					
	everyday	0	0.0	0	0.0
	4–6 times a week	0	0.0	0	0.0
	2–3 times a week	2	3.2	1	1.1
	once a week	3	4.8	2	2.2
	once every 2 weeks	6	9.7	7	7.5
	never	45	72.6	68	73.1
	no data	6	9.7	15	16.1
Almonds					
	everyday	3	4.8	3	3.2
	4–6 times a week	2	3.2	1	1.1
	2–3 times a week	4	6.5	4	4.3
	once a week	5	8.1	3	3.2
	once every 2 weeks	13	21.0	13	14.0
	never	29	46.8	54	58.1
	no data	6	9.7	15	16.1
Peanuts					
	everyday	1	1.6	1	1.1
	4–6 times a week	1	1.6	1	1.1
	2–3 times a week	1	1.6	1	1.1
	once a week	1	1.6	2	2.2
	once every 2 weeks	2	3.2	4	4.3
	never	50	80.6	69	74.2
	no data	6	9.7	15	16.1

**Table A5:** Eggs consumption among investigated patients.

		Supplemented *n* = 62		Non-Supplemented *n* = 93	
Eggs		*n*	%	*n*	%
	everyday	4	6.5	7	7.5
	4–6 times a week	1	1.6	1	1.1
	2–3 times a week	25	40.3	22	23.7
	once a week	23	37.1	32	34.4
	once every 2 weeks	1	1.6	13	14.0
	never	3	4.8	3	3.2
	no data	5	8.1	15	16.1

**Table A6:** Flakes, cereals, and seeds consumption among investigated patients.

		Supplemented *n* = 62		Non-Supplemented *n* = 93	
Oatmeal		*n*	%	*n*	%
	everyday	6	9.7	1	1.1
	4–6 times a week	4	6.5	6	6.5
	2–3 times a week	14	22.6	7	7.5
	once a week	10	16.1	12	12.9
	once every 2 weeks	4	6.5	5	5.4
	never	18	29.0	47	50.5
	no data	6	9.7	15	16.1
Cornflakes					
	everyday	4	6.5	5	5.4
	4–6 times a week	1	1.6	3	3.2
	2–3 times a week	10	16.1	3	3.2
	once a week	2	3.2	4	4.3
	once every 2 weeks	1	1.6	2	2.2
	never	38	61.3	61	65.6
	no data	6	9.7	15	16.1
Rice flakes					
	everyday	0	0.0	0	0.0
	4–6 times a week	0	0.0	0	0.0
	2–3 times a week	1	1.6	0	0.0
	once a week	2	3.2	0	0.0
	once every 2 weeks	0	0.0	0	0.0
	never	53	85.5	78	83.9
	no data	6	9.7	15	16.1
Sunflower seeds					
	everyday	3	4.8	1	1.1
	4–6 times a week	2	3.2	1	1.1
	2–3 times a week	2	3.2	2	2.2
	once a week	4	6.5	6	6.5
	once every 2 weeks	7	11.3	6	6.5
	never	38	61.3	62	66.7
	no data	6	9.7	15	16.1
Pumpkin seeds					
	everyday	2	3.2	0	0.0
	4–6 times a week	1	1.6	1	1.1
	2–3 times a week	0	0.0	1	1.1
	once a week	3	4.8	4	4.3
	once every 2 weeks	4	6.5	4	4.3
	never	46	74.2	68	73.1
	no data	6	9.7	15	16.1
Wheat bread					
	everyday	6	9.7	5	5.4
	4–6 times a week	1	1.6	1	1.1
	2–3 times a week	1	1.6	1	1.1
	once a week	0	0.0	0	0.0
	once every 2 weeks	1	1.6	1	1.1
	never	47	75.8	69	74.2
	no data	6	9.7	16	17.2
Rye bread					
	everyday	21	33.9	18	19.4
	4–6 times a week	4	6.5	6	6.5
	2–3 times a week	1	1.6	3	3.2
	once a week	1	1.6	2	2.2
	once every 2 weeks	2	3.2	0	0.0
	never	27	43.5	49	52.7
	no data	6	9.7	15	16.1
Whole wheat bread					
	everyday	17	27.4	15	16.1
	4–6 times a week	9	14.5	7	7.5
	2–3 times a week	2	3.2	7	7.5
	once a week	2	3.2	6	6.5
	once every 2 weeks	1	1.6	3	3.2
	never	25	40.3	39	41.9
	no data	6	9.7	16	17.2

**Table A7:** Selected vegetables, fruits, tofu, and diet supplementation among investigated patients.

		Supplemented *n* = 62		Non-Supplemented *n* = 93	
Avocado		*n*	%	*n*	%
	everyday	1	1.6	0	0.0
	4–6 times a week	0	0.0	0	0.0
	2–3 times a week	2	3.2	0	0.0
	once a week	3	4.8	3	3.2
	once every 2 weeks	4	6.5	3	3.2
	never	46	74.2	72	77.4
	no data	6	9.7	15	16.1
Lettuce					
	everyday	5	8.1	10	10.8
	4–6 times a week	1	1.6	1	1.1
	2–3 times a week	17	27.4	15	16.1
	once a week	12	19.4	11	11.8
	once every 2 weeks	4	6.5	13	14.0
	never	17	27.4	28	30.1
	no data	6	9.7	15	16.1
Spinach					
	everyday	0	0.0	0	0.0
	4–6 times a week	0	0.0	1	1.1
	2–3 times a week	3	4.8	4	4.3
	once a week	13	21.0	7	7.5
	once every 2 weeks	11	17.7	13	14.0
	never	29	46.8	53	57.0
	no data	6	9.7	15	16.1
Tofu					
	everyday	0	0.0	0	0.0
	4–6 times a week	0	0.0	0	0.0
	2–3 times a week	1	1.6	0	0.0
	once a week	0	0.0	0	0.0
	once every 2 weeks	1	1.6	0	0.0
	never	54	87.1	78	83.9
	no data	6	9.7	15	16.1
Vitamin supplementation					
	yes	52	83.9	47	50.5
	no	10	16.1	46	49.5
	no data	0	0.0	0	0.0
Potato fries					
	yes	21	33.9	29	31.2
	no	38	61.3	55	59.1
	no data	3	4.8	9	9.7
Chips					
	yes	14	22.6	16	17.2
	no	45	72.6	68	73.1
	no data	3	4.8	9	9.7
